# Validation of Ten Noninvasive Diagnostic Models for Prediction of Liver Fibrosis in Patients with Chronic Hepatitis B

**DOI:** 10.1371/journal.pone.0144425

**Published:** 2015-12-28

**Authors:** Jieyao Cheng, Jinlin Hou, Huiguo Ding, Guofeng Chen, Qing Xie, Yuming Wang, Minde Zeng, Xiaojuan Ou, Hong Ma, Jidong Jia

**Affiliations:** 1 Liver Research Center, Beijing Friendship Hospital, Capital Medical University, Beijing Key Laboratory of Translational Medicine in Liver Cirrhosis & National Clinical Research Center of Digestive Diseases, Beijing, China; 2 Hepatology Department, Nanfang Hospital, Southern Medical University, Guangzhou, China; 3 Gastroenterology and Hepatology Department, Beijing Youan Hospital, Capital Medical University, Beijing, China; 4 Diagnosis and Treatment Center of Liver Fibrosis, 302 Hospital of the China, Beijing, China; 5 Infectious Diseases Department, Ruijin Hospital, Shanghai Jiao Tong University, Shanghai, China; 6 Infectious Diseases Department, Southwest Hospital, Third Military Medical University, Chongqing, China; 7 Gastroenterology Department, Renji Hospital, Shanghai Jiao Tong University, Shanghai, China; Yonsei University College of Medicine, REPUBLIC OF KOREA

## Abstract

**Background and Aims:**

Noninvasive models have been developed for fibrosis assessment in patients with chronic hepatitis B. However, the sensitivity, specificity and diagnostic accuracy in evaluating liver fibrosis of these methods have not been validated and compared in the same group of patients. The aim of this study was to verify the diagnostic performance and reproducibility of ten reported noninvasive models in a large cohort of Asian CHB patients.

**Methods:**

The diagnostic performance of ten noninvasive models (HALF index, FibroScan, S index, Zeng model, Youyi model, Hui model, APAG, APRI, FIB-4 and FibroTest) was assessed against the liver histology by ROC curve analysis in CHB patients. The reproducibility of the ten models were evaluated by recalculating the diagnostic values at the given cut-off values defined by the original studies.

**Results:**

Six models (HALF index, FibroScan, Zeng model, Youyi model, S index and FibroTest) had AUROCs higher than 0.70 in predicting any fibrosis stage and 2 of them had best diagnostic performance with AUROCs to predict F≥2, F≥3 and F4 being 0.83, 0.89 and 0.89 for HALF index, 0.82, 0.87 and 0.87 for FibroScan, respectively. Four models (HALF index, FibroScan, Zeng model and Youyi model) showed good diagnostic values at given cut-offs.

**Conclusions:**

HALF index, FibroScan, Zeng model, Youyi model, S index and FibroTest show a good diagnostic performance and all of them, except S index and FibroTest, have good reproducibility for evaluating liver fibrosis in CHB patients.

**Registration Number:**

ChiCTR-DCS-07000039.

## Introduction

Chronic hepatitis B (CHB) is a major global health problem, which can lead to cirrhosis, decompensation and hepatocellular carcinoma (HCC). The recent guidelines[1] on the management of CHB have proposed that the presence of significant fibrosis and cirrhosis are indication for treatment and close monitoring for complications of portal hypertension and development of HCC. Therefore, assessment of liver fibrosis in patients with CHB is of paramount importance to predict disease progression, determine the optimal timing and evaluate the efficacy of antiviral therapy.

At present, liver biopsy remains the gold standard for assessing liver fibrosis. However, liver biopsy is an invasive procedure with a potential risk of complications, especially in those with advanced fibrosis and cirrhosis, and its diagnostic accuracy is compromised by sampling error as well as interobserver variations[2–4].

Therefore, noninvasive methods for assessing liver fibrosis have been the focus of translational research. Noninvasive models such as aspartate aminotransferase (AST)-to-platelet ratio index (APRI)[5], FibroTest[6], FibroScan[7], Zeng model[8] and so on, comprising various biochemical and clinical parameters have been derived from patients with CHB, CHC and alcoholic liver disease. Most of these studies reported good values in AUROC analysis but have not been externally validated and compared in the same group patients. Therefore, in the present study we validated the diagnostic performance and evaluated the reproducibility of these forementioned models against liver histology in a big cohort of Chinese patients with CHB.

## Methods

### Patients

Between September 2007 and April 2009, patients with CHB who underwent a percutaneous liver biopsy at the seven hospitals (Beijing Friendship Hospital, Beijing; Beijing Youan Hospital, Beijing; 302 Hospital of the Chinese People's Liberation Army, Beijing; Nanfang Hospital, Guangzhou; Ruijin Hospital, Shanghai; Renji Hospital, Shanghai; Southwest Hospital, Chongqing) who met the following criteria were recruited into this study. (I) Age between 18 and 65 years; (II) hepatitis B surface antigen (HBsAg) positive for longer than 6 months; (III) at least two weeks off-therapy of biofendate or biocyclol before enrollment; (IV) written informed consent. Exclusion criteria included: (I) white blood cell count <3.5×10^9^/L, or platelet count <80×10^9^/L, or prothrombin index <60%; (II) evidence of a co-infection with hepatitis C; (III) evidence of any other acquired or inherited liver disease; (IV) a history of decompensated cirrhosis defined as jaundice in the presence of cirrhosis, ascites, bleeding gastric or esophageal varices or encephalopathy; (V) a history of malignancies including hepatocellular carcinoma; (VI) lactation; (VII) body mass index (BMI) > 28 kg/m^2^; (VIII) cardiac pacemaker or defibrillator carrier; (IX) unhealed wound in right upper quadrant.

All patients wrote informed consent before inclusion. This study was approved by ethics committee of principle investigator, i.e. Beijing Friendship Hospital, for all the participating centers in 2007, in accordance with the guidelines of the 1975 Declaration of Helsinki.

### Liver Histology

Needle liver biopsy specimens were obtained with a 16-gauge needle under ultrasound guidance. To be considered as adequate for scoring, the liver biopsies had to measure at least 10mm and contain 8 portal tracts. All the liver biopsy specimens were routinely processed by formalin fixation, paraffin-embedding, and sectioned at 5μm thickness, and then stained with Masson Trichrome and reticulin staining for histological assessment. All specimens were assessed by two independent pathologists blinded to patient clinical and laboratory characteristics. Fibrosis was scored on a 5-point scale according to the METAVIR scoring system[2, 9]: F0, no fibrosis; F1, portal fibrosis without septa; F2, portal fibrosis with few septa; F3, numerous septa without cirrhosis; F4, cirrhosis. Discordant cases were reviewed by both pathologists together to reach consensus.

### Liver Stiffness Measurement

Liver stiffness measurement (LSM) were performed using the FibroScan® medical device (Echosens, Paris, France). LSM was performed by well-trained operators on the same day as liver biopsy. The procedure was based on at least ten validated measurements. The success rate was calculated as the number of measurements. The median value, expressed in kilopascals (kPa), was considered representative of the liver stiffness value. The liver stiffness value was considered reliable only if at least 10 successful acquisitions were obtained, the overall success rate was ≥ 60%, and the interquartile range over median value of liver stiffness was ≤ 30%.

### Sample collection

Blood samples were collected in evacuated tubes (Monovette 02.1063, Sarstedt, Germany), allowed to clot for 30 min at room temperature and centrifuged at 1600g for 15 min at 4°C. Sera were frozen at -80°C within 2 h after collection.

### Biochemical Measurements

Enzyme activities (U/L) were measured in each hospital by a Modular P800 automatic analyzer using standard methods (Roche Diagnostics, Mannheim, Germany): alanine aminotransferase (ALT: female <34, male <45), aspartate aminotransferase (AST: female <31, male <35), alkaline phosphatase (ALP: female 35–104, male 40–129), gamma-glutamyltransferase (GGT: female <38, male <55). Serum albumin (normal range 35–52 g/L) was routinely measured immunonephelometrically using the Modular P 800 (Roche Diagnostics, Mannheim, Germany). 8mL additional serum and 3mL plasma were taken around the same time (stored at -80°C) and send to a central laboratory (302 Hospital of the Chinese People's Liberation Army) for fibrosis markers analysis. Haptoglobin (HPT), alpha2-macroglobulin (α2-M) and apolipoprotein A (apoA1) were measured with immunoturbidimetry (Beckman Coultery, USA). PIIINP and hyaluronic Acid (HA) were measured with chemiluminescence immunoassay (Xiamen Lujia Biotechnology Company reagents, China), and TIMP-1 and TGFβ1 were measured with ELISA (R&D Systems reagents, USA).

### Noninvasive models

Up to October 2013, there are serval published noninvasive models for assessing liver fibrosis. Most of them are derived from CHC patients, including APRI[5], FibroTest[6] and FIB-4[10], which are the most well validated noninvasive models. Fourteen models derived from CHB patients including Zeng model, Hui model, HALF index, S index, Youyi model, APAG, FibroScore, Cirrhosis Score (CS), Compensated Cirrhosis Index (CCI), Cirrhosis Index (CI), Mohamadnejad model, SPRI and ASPRI [8, 11–21]. In the fourteen models, seven models were excluded for the follow reasons: (i) incomplete ultrasound information (SPRI, ASPRI, CCI and CI were excluded), (ii) inequal lower detection limit of HBV-DNA (Mohamadnejad model was excluded), (iii) patients less than 150 in training group (CS and FibroScore were excluded). Therefore, seven CHB models and three well-validated CHC models were evaluated in this study, including APRI, FibroTest, FIB-4, FibroScan, Zeng model, Hui model, HALF index, S index, Youyi model and APAG. The scores were calculated directly by the instrument employing the equations according to the original articles which are listed in Table 1.

**Table 1:** The formulas of the ten noninvasive models.

Models	Formulas
APRI[5]	AST (/ULN) ×100 / PLT (10^9^/L)
FibroTest[6]	The scores were calculated through the website (www.biopredictive.com).
FIB-4[10]	Age ×AST(U/L) / [PLT(10^9^/L)×(ALT (U/L^)1/2^]
FibroScan[7]	Liver stiffness measure, LSM
Zeng model[8]	-13.995+3.220×log(α2-M)+3.096×log(age)+2.254×log(GGT)+2.437× log(HA)
Hui model[11]	expD/(1+expD), D = 3.148 + 0.167×BMI +0.088×TBIL- 0.151×ALB-0.019×PLT
HALF index[12]	(-0.017×HPT)- (0.022×apoA1) +(0.012×α2-M)+(0.691×LSM)
S Index[13]	1000×GGT / (PLT×ALB^2^)
Youyi model[14]	10×e^D^ / (l+e^D^), D = -6.29 + l.678×ln(age) - l.786×ln(PLT) + 1.177×ln(GGT) + 1.019×ln (HA)
APAG[15]	e^P^/ (1+e^P^), P = -9.340 + 0.997×ln(age) + 6.355×ln(PT) - 3.372×ln(ALB(g/L)) + 0.677 × ln(GGT(U/L))

### Statistical Analysis

The diagnostic performance of each noninvasive model was assessed using receiver operating characteristic curves (ROC). The areas under the ROC curves (AUROC) as well as 95% confidential interval (CI) of AUROC were calculated. AUROC values for different diagnostic criteria for the same data set were compared with the De Long method [22], using Medcalc Software version 12.2.1.0 (Medcalc, Mariakerke, Belgium). A *P*-value < 0.05 was considered statistically significant.

To evaluate the reproducibility of these models, we recalculated their diagnostic values (Sen, Spe, PPV, NPV, positive likelihood ratios (LR+) and negative likelihood ratios (LR-) at the given cut-off values defined by the original studies [5–8, 10–15]. Statistical analysis was performed by SPSS software version 17.0 (SPSS, Chicago, IL, USA).

## Results

### Patient characteristics

**Fig 1:**
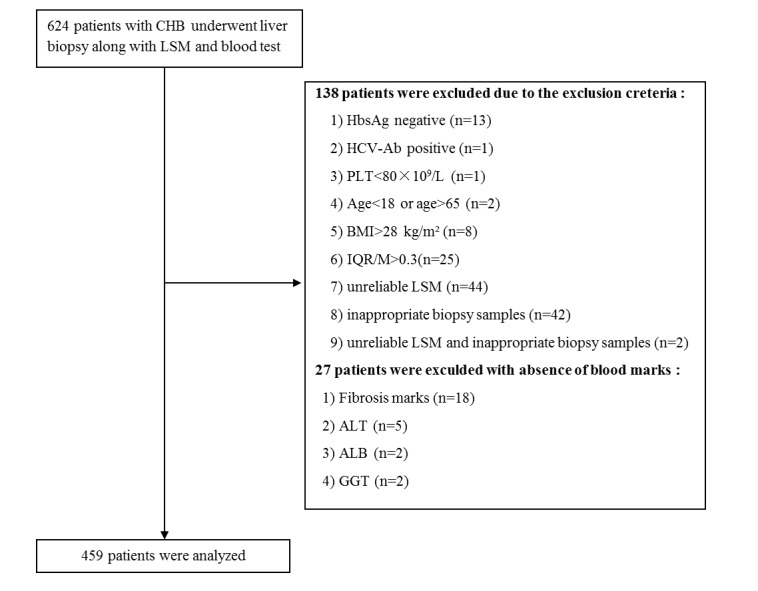
Flow chart describing the selection of the study population. 459 subjects were finally recruited for analysis.

From September 2007 to April 2009, we included 624 CHB patients for this study. Of these, 138 patients were excluded base on exclusion criteria, and 27 patients were excluded because of the incomplete serum analysis data. So, 459 patients who met the eligibility criteria were used as study subjects for the following analysis, as shown in Fig 1.

Patient characteristics are summarized in Table 2. The median BMI was 22.0 kg/m^2^. The median PLT was 168.0×10^9^/L. The median ALT and AST level were 50.4 U/L and 36.0 U/L, respectively. Patient distribution for METAVIR and Ishak fibrosis stage was as follows: F0, n = 37; F1, n = 142; F2, n = 145; F3, n = 65; F4, n = 70. The values of ten noninvasive models were significant increased in patients with significant fibrosis (F2/F3/F4) vs the patients with no significant fibrosis (F0/F1), as shown in Table 3.

**Table 2:** Characteristics of the 459 Patients.

Characteristics	Value (N = 459)
**Demographic data**	
Age (years)	33 (26~43)
Male gender, no. (%)	341 (74.3)
**Laboratory data**	
BMI (kg/m^2^)	22.0 (20.3~23.9)
PLT (10^9^/L)	168.0 (124.0~210.0)
PT (s)	12.2 ± 1.4
ALB (mg/dL)	45.0 (41.4~48.3)
ALT (U/L)	50.4 (27.0~108.0)
AST (U/L)	36.0 (25.0~67.0)
GGT (U/L)	27.0 (17.0~57.0)
TBIL (μmol/L)	34.0 (22.0~53.0)
HA (μg/L)	61.59 (41.34~139.8)
PIIINP (μg/L)	86.88 (28.12~196.00)
TIMP-1 (μg/L)	193.50 (132.85~292.95)
α2-M (mg/dL)	239.0 (194.0~298.0)
HPT (mg/dL)	40.8 (24.6~67.1)
apoA1 (g/L)	1.5 ± 0.3
**Liver stiffness (kPa)**	6.8 (5.1~11.7)
**HALF index**	7.0 (5.4~10.4)
**Zeng model**	6.0 (5.2~7.4)
**S index**	0.09 (0.05~0.22)
**Hui model**	0.13 (0.04~0.36)
**Youyi model**	2.25 (0.80~5.56)
**APAG**	0.36 (0.22~0.62)
**APRI**	0.61 (0.37~1.10)
**FibroTest**	0.38 (0.23~0.63)
**FIB-4**	1.15 (0.73~2.01)
**Fibrosis stage, no. (%)**	
■ F0	37 (8.1)
■ F1	142 (30.9)
■ F2	145 (31.6)
■ F3	65 (14.2)
■ F4	70 (15.2)

NOTE. Quantitative variables are expressed as mean ± SD for normal distribution, and median (P25, P75) for abnormal distribution. Categoric variables are expressed as frequency (percentages).

**Table 3:** Ten Models Associated With Presence of Significant Fibrosis (Stages 2–4) in All Patients.

Models	No significant fibrosis (F0-F1) (N = 179)	Significant fibrosis (F2-F4) (N = 280)	*P* Value (Univariate)
**Liver stiffness (kPa)**	5.3(4.4~6.5)	9.2 (6.2~15.9)	<0.01
**HALF index**	5.4 (4.4~6.6)	9.1 (6.5~13.5)	<0.01
**Zeng model**	3.3 (2.7~4.2)	4.5 (3.7~6.0)	<0.01
**S index**	0.05 (0.03~0.09)	0.15 (0.07~0.32)	<0.01
**Hui model**	0.08 (0.03~0.20)	0.19 (0.06~0.45)	<0.01
**Youyi model**	1.07 (0.49~3.07)	3.57 (1.36~7.28)	<0.01
**APAG**	0.26 (0.15~0.42)	0.47 (0.26~0.68)	<0.01
**APRI**	0.45 (0.30~0.70)	0.79 (0.48~1.46)	<0.01
**FibroTest**	0.26 (0.17~0.40)	0.50 (0.29~0.74)	<0.01
**FIB-4**	0.91 (0.57~1.42)	1.43 (0.87~2.37)	<0.01

### Diagnostic performance of noninvasive models in comparison with liver biopsy

**Fig 2:**
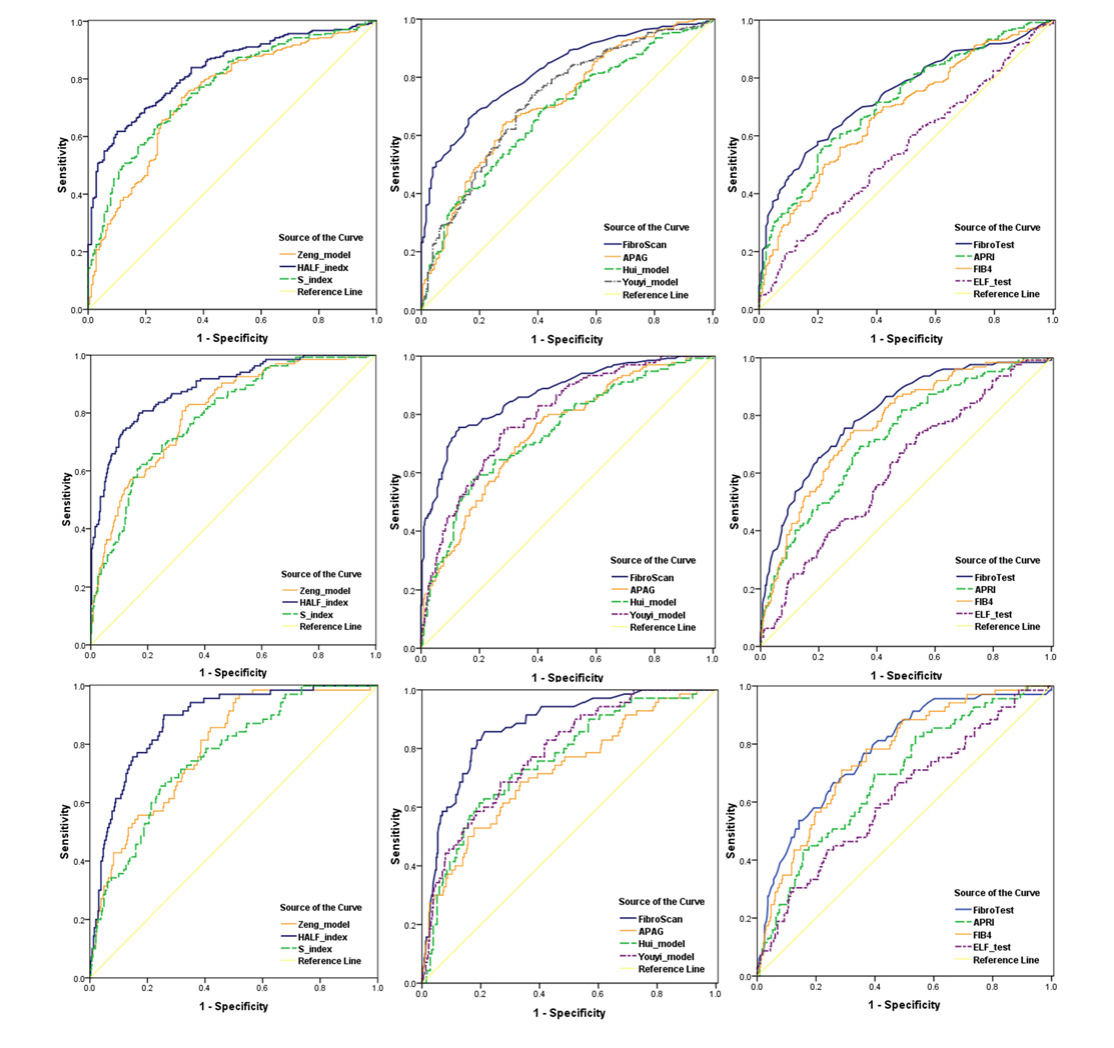
Receiver operating characteristic (ROC) curves for noninvasive models in the diagnosis of significant fibrosis (F2-4, A), advanced fibrosis (F3-4,B), and cirrhosis (F4, C).

Diagnostic performances of the noninvasive models were evaluated by AUROCs determined for the whole population according to their histology fibrosis stages, which were shown in Table 4. For significant fibrosis (≥F2), the AUROCs of HALF index, Zeng model, FibroScan, Youyi model, S index and FibroTest were varies from 0.70 to 0.90, while the AUROCs of Hui model, APAG, APRI and FIB-4 were less than 0.70 (Fig 2A). For advanced fibrosis (Fig 2B) and cirrhosis (Fig 2C), the AUROCs of all models were better than 0.70, except APRI.

**Table 4:** AUROC and 95%CI for each model, according to METAVIR Fibrosis Stages.

Models	F ≥ 2	F ≥ 3	F ≥ 4
HALF index	**0.83 (0.79, 0.86)**	**0.89 (0.85, 0.91)**	**0.89 (0.85, 0.91)**
Zeng model	**0.74 (0.70, 0.78)**	**0.81 (0.77, 0.84)**	**0.79 (0.75, 0.82)**
S index	**0.77 (0.73, 0.81)**	**0.80 (0.76, 0.84)**	**0.77 (0.73, 0.81)**
FibroScan	**0.82 (0.78, 0.85)**	**0.87 (0.84, 0.90)**	**0.87 (0.84, 0.90)**
Hui model	0.68 (0.63, 0.72)	**0.74 (0.70, 0.78)**	**0.76 (0.72, 0.80)**
Youyi model	**0.73 (0.70, 0.76)**	**0.80 (0.76, 0.84)**	**0.78 (0.74, 0.82)**
APAG	0.69 (0.66, 0.73)	**0.74 (0.70, 0.78)**	**0.72 (0.68, 0.76)**
APRI	0.69 (0.66, 0.73)	**0.73 (0.68, 0.77)**	0.69 (0.64, 0.74)
FibroTest	**0.74 (0.70, 0.78)**	**0.81 (0.77, 0.84)**	**0.78 (0.74, 0.82)**
FIB-4	0.68 (0.63, 0.72)	**0.77 (0.73, 0.81)**	**0.76 (0.72, 0.80)**

The ROC curves of respective noninvasive models had been compared with each other using the Delong method (S1 Fig and S1 Table). HALF index and FibroScan showed significantly better performances for diagnosis of significant fibrosis, advanced fibrosis and cirrhosis than any other serum models (all *P*<0.05). The accuracy of FibroScan and HALF index for prediction of ≥F2, ≥F3 and F4 was statistically equivalent to each other (*P* = 0.25 for ≥F2, *P* = 0.30 for ≥F3, *P* = 0.45 for F4).

The AUROCs for F≥2, F≥3 and F4 of HALF index, FibroScan, Zeng model, Youyi model, S index and FibroTest were consistently better than 0.70, so the six noninvasive models were selected for further evaluation.

### Evaluation the reproducibility of the six noninvasive models

The given cutoff values for each model obtained from the original articles[6–8, 12–14] were listed in Table 5. For FibroScan, the diagnostic accuracy for F≥2, F≥3 and F4 were 73%, 84% and 88% respectively, which were similar to the values of original article[7] (76%, 90% and 94% for F2, F3 and F4), indicating FibroScan has a good reproducibility to predict fibrosis stage in CHB patients. The diagnostic accuracy of Youyi model were 65%, 71% and 77% with the given cutoff values in this study, similar to the values of original article[14] (79%, 82% and 77% for F2, F3 and F4). Since low cutoffs were originally described to rule out significant fibrosis, attention must be paid to NPV. The NPV for HALF index was 100% with cutoff value < 2.22, higher than the one in original article[12] (NPV 94.7%). While high cutoffs were described to confirm significant fibrosis, attention should be paid to PPV. The PPV for HALF index was 95% which was similar to the one in original article[12] (PPV 100%). These results demonstrated that HALF index has a good reproducibility. Compared to the original article[8] (NPV 86.1% at value <3.0, PPV 91.1% at value >8.7), Zeng model also has a good reproducibility. Whereas the NPV of S index in this study with low cutoff value (less than 0.1) was only 56%, less than the one of the original article[13] (NPV 65.57%). The NPV of FibroTest was 59% at the cutoff value 0.1 in this study compared with 100% in the original article[6], which means the reproducibility of FibroTest is not good enough.

**Table 5:** Diagnostic values of FibroScan, Youyi model, Zeng model, HALF index, S index and FibroTest with their given cutoff values in predicting liver fibrosis in CHB patients.

Models	Fibrosis stage	cutoffs	Sen	Spe	AC	PPV	NPV	LR+	LR-
FibroScan[7]	F≥2	7.2(MA)	0.66	0.83	**0.73**	0.86	0.61	3.94	0.41
	F≥3	10.5(MA)	0.70	0.90	**0.84**	0.75	0.88	7.28	0.33
	F = 4	18.2(MA)	0.49	0.95	**0.88**	0.62	0.91	9.80	0.54
Youyi model [14]	F≥2	2.2	0.63	0.68	**0.65**	0.75	0.54	1.95	0.54
	F≥3	3.0	0.76	0.69	**0.71**	0.50	0.87	2.45	0.35
	F = 4	5.4	0.59	0.80	**0.77**	0.34	0.91	2.92	0.51
Zeng Model [8]	F≥2	Low cutoff <3.00	0.89	0.38	0.69	0.69	**0.68**	1.43	0.29
		High cutoff >8.70	0.02	1.00	0.40	**1.00**	0.39	-	0.98
HALF index [12]	F≥2	Low cutoff <2.22	1.00	0.02	0.62	0.61	**1.00**	1.02	0
		High cutoff >7.23	0.39	0.97	0.63	**0.95**	0.50	13.00	0.63
S index [13]	F≥2	Low cutoff <0.10	0.61	0.77	0.67	0.81	**0.56**	2.65	0.51
		High cutoff >0.50	0.16	0.99	0.48	**0.96**	0.43	14.06	0.85
FibroTest [6]	F≥2	Low cutoff <0.10	0.97	0.06	0.61	0.61	**0.59**	1.03	0.47
		High cutoff >0.60	0.41	0.93	0.62	**0.90**	0.51	5.88	0.63

NOTE. The given cutoff values obtained from the original articles[6–8, 12–14]. Sen, sensitivity; Spe, specificity; AC, Accuracy; PPV, positive predictive value; NPV, negative predictive value; LR+, positive likelihood ratio; LR-, negative likelihood ratio.

## Discussion

In this study, we compared and verified ten noninvasive fibrosis evaluation models in diagnostic performance and reproducibility in the same group of CHB patients. Among the ten models, there are six models (HALF index, FibroScan, Zeng model, Youyi model, S index and FibroTest) consistently show an AUROC over 0.70 in diagnosing significant fibrosis, advanced fibrosis and cirrhosis, especially HALF index and FibroScan have a much better accuracy in diagnosing any stage of liver fibrosis. Four models, HALF index, FibroScan, Zeng model and Youyi model, have a much better reproducibility than the other six models.

The first important finding of this study was that models containing of imaging techniques or direct serum markers (the HALF index, FibroScan, Zeng model, and Youyi model) had better diagnostic values for CHB patients than those only containing of indirect serum markers (the S index, Hui model, APAG, APRI and FIB-4), which was similar to the previous validation studies[13, 23]. Secondly, noninvasive models derived from CHC are not useful tools to assess liver fibrosis in patients with CHB because of the different pathogenesis and histology progression of fibrosis between CHB and CHC. Thirdly, LSM alone showed similar diagnostic performance to the complex models that combine LSM and other serum parameters (such as HALF index).

This study had two unique features. The first one is that most noninvasive models derived from CHB only validated in an internal validation cohort from the same population with the training cohort or validated in a single-center external cohort[23], while our study validated ten reported noninvasive models (seven derived from CHB patients and three from CHC patients) in a large multicenter cohort from different parts of China. The large number and geographic diversity of patients played a key role in validating the reproducibility. The other is that we recruited patients who underwent not only blood tests but also LSM, and this allowed us to compare the diagnostic performance of these two major categories of noninvasive diagnostic modalities in Asian populations with CHB[24–26].

This study did have some limitations. Firstly, we recruited only patients with liver biopsy specimens at least 1 cm in length and 8 complete portal tracts, which could satisfy the primary assessment of liver fibrosis, but still not fulfill the more stringent criterion recommended by the guideline of AASLD[3]. Secondly, in our study 39% patients had fibrosis stage < F2, and less patients (15%) had cirrhosis, which may lead to a selection bias. Thirdly, since patients enrolled from 7 different hospitals, FibroScan was performed by the different operator and potentially subject to interobserver variability. To reduce this interobserver variability, we setup standard operating procedures for FibroScan when designing the experiments, and all the operators had been trained together before this study. Finally, this is a cross-sectional study. Whether these models can be used to assess treatment response and long term clinical outcome in CHB patients still needs prospective cohort study.

In conclusion, in a big cohort of CHB patients we found that six noninvasive models including HALF index, FibroScan, Zeng model, Youyi model, S index and FibroTest had a good diagnosis accuracy and four of them (HALF index, FibroScan, Zeng model and Youyi model) had a good reproducibility for evaluating liver fibrosis in CHB patients.

## Supporting Information

S1 Appendix Ethics consent. (ZIP)

S2 Appendix Database. (XLS)

S1 Fig AUROC of ten noninvasive models in the diagnosis of significant fibrosis (F2-4), advanced fibrosis (F3-4), and cirrhosis (F4). (TIF)

S1 Table AUROC of respective models was compared with each other and the *P* values was listed. (DOCX)

## Abbreviations

CHB: chronic hepatitis B
LSM: liver stiffness measurement
AUROC: area under receiver operating characteristic curve
CI: confidence interval
MS: maximum sum of sensitivity and specificity
Sen: sensitivity, Spe, specificity
AC: accuracy
PPV: positive predictive value
NPV: negative predictive value
LR+: positive likelihood ratio
LR-: negative likelihood ratio
ULN: upper limit of normal
