# Supplementary material for: Validation of Ten Noninvasive Diagnostic Models for Prediction of Liver Fibrosis in Patients with Chronic Hepatitis B
Source: PLoS One. 2015 Dec 28;10(12):e0144425. doi: 10.1371/journal.pone.0144425 (PMC4692502; doi:10.1371/journal.pone.0144425)
Supplement: S1 Appendix — (ZIP) [file pone.0144425.s001.zip › Ethics consent-Beijing Friendship Hospital.pdf]

北京友谊医院国家药品临床研究基地

伦理委员会设备、仪器临床试验审批表

（2007）设备、仪器临审第 26 号

实验项目名称	FIBROSCAN 无创评价慢性乙型肝炎肝纤维化及肝硬化程度				
试验目的	临床验证			例数	100 例
申请科室	肝病中心	承担责任	组织　参与√	项目负责人	贾继东
医学伦理委员会成员：					
姓　名	职　务	同意	不同意	签名	日期
魏　玫	主任委员	①		[illegible]	2007.12.5
刘　建	副主任委员	①		[illegible]	2007.12.5
王汝龙	副主任委员	①		[illegible]	2007.12.5
韩小茜	委员	①		[illegible]	2007.12.5
王　宇	委员	①		[illegible]	2007.12.5
王质刚	委员	①		[illegible]	2007.12.5
沈潞华	委员	①		[illegible]	2007.12.5
张淑文	委员	①		[illegible]	2007.12.5
冯　捷	委员	①		[illegible]	2007.12.5
蔡　真	委员	①		[illegible]	07.12.5

审批意见：①同意√；②作必要的修改后同意；③不同意；④终止或暂停已批准的试验。

首都医科大学附属北京友谊医院医学伦理委员会

主任委员 [illegible]

签名

2007 年 12 月 5 日

声明：参加审批的伦理委员会独立于医院和申办者，并且本伦理委员会严格按照中国 GCP 及相关法规组成和工作。

伦理委员会地址：北京市宣武区永安路 95 号（邮编：100050）

北京友谊医院伦理委员会

关于设备、仪器临床试验伦理要求

（2007）设备、仪器临审第26 号

<table>
<tr><td>试验项目名称</td><td colspan="5">FIBROSCAN 无创评价慢性乙型肝炎肝纤维化及肝硬化程度</td></tr>
<tr><td>试验目的</td><td colspan="3">临床验证</td><td>例数</td><td>100 例</td></tr>
<tr><td>申请科室</td><td>肝病中心</td><td>承担责任</td><td>参与</td><td>项目负责人</td><td>贾继东</td></tr>
<tr><td>研制单位</td><td colspan="5">法国 ECHOSENS 公司</td></tr>
<tr><td>试验申办者</td><td colspan="5">中国肝炎防治基金会王宝恩肝纤维化基金</td></tr>
<tr><td rowspan="2">报送材料</td><td>政府部门批件</td><td>无需政府批件</td><td rowspan="2">审查意见</td><td>研究者资格</td><td>符合要求</td></tr>
<tr><td>检验报告</td><td>有</td><td>试验方案</td><td>适当</td></tr>
<tr><td>受试对象</td><td colspan="5">慢性乙型肝炎肝纤维化及早期肝硬化患者</td></tr>
<tr><td>适应症或功能</td><td colspan="5">慢性乙型肝炎肝纤维化及肝硬化</td></tr>
<tr><td>临床评价标准</td><td colspan="5">按照统计学标准进行操作</td></tr>
<tr><td>安全性及风险</td><td colspan="5">可能发生的副作用：无
可能发生的器械不良反应：无
有损伤检查项目及次数：无
国内外是否用于人体试验：国外已用于临床检查</td></tr>
<tr><td>知情情况</td><td colspan="5">签署知情同意书</td></tr>
<tr><td>被试验者受益</td><td colspan="5">和本研究有关的检查和化验免费，并提供患者交通补贴。</td></tr>
<tr><td>其它</td><td colspan="5"></td></tr>
</table>
