# Supplementary material for: Validation of Ten Noninvasive Diagnostic Models for Prediction of Liver Fibrosis in Patients with Chronic Hepatitis B
Source: PLoS One. 2015 Dec 28;10(12):e0144425. doi: 10.1371/journal.pone.0144425 (PMC4692502; doi:10.1371/journal.pone.0144425)
Supplement: S1 Appendix — (ZIP) [file pone.0144425.s001.zip › Ethics consent-Renji Hospital.pdf]

上海交通大学医学院附属仁济医院伦理委员会
临床药物试验审批件

仁济伦审[2007] 56 号

<table>
<tr><td>试验项目名称</td><td colspan="6">FIBROSCAN 无创评价慢性肝炎肝纤维化及肝硬化程度研究方案</td></tr>
<tr><td>药物名称</td><td>FIBROSCAN
超声诊断仪</td><td>剂型</td><td>医疗器械</td><td>SFDA 号</td><td colspan="2"></td></tr>
<tr><td>申办单位</td><td colspan="6">中国肝炎防治基金会</td></tr>
<tr><td rowspan="2">临床试验科室
（专业）</td><td rowspan="2">消化科</td><td colspan="2">项目负责人</td><td>曾民德</td><td>职称</td><td>主任医师</td></tr>
<tr><td colspan="2">主要参加者</td><td>茅益民</td><td>职称</td><td>副主任医师</td></tr>
<tr><td>其他参加单位</td><td colspan="6">首都医科大学附属北京友谊医院　中国人民解放军第 302 医院
首都医科大学附属北京佑安医院　上海交通大学医学院附属仁济医院
第三军医大学附属重庆市西南医院　南方医科大学南方医院</td></tr>
<tr><td>报送材料</td><td colspan="6">☐ 国家食品药品监督管理局批号：无
☐ 药品检验报告：国家食品药品监督管理局武汉医疗器械质量监督检验中心 2007220
☐ 院外伦理审查批件：　无
☐ 临床试验方案及日期：2007 年 8 月
☐ 知情同意书及日期：　有
☐ 研究者手册：2007 年 8 月
☐ 参考文献或其他：委托书、研究者简历、用户手册、产品标准、CRF</td></tr>
<tr><td>伦理委员会意见</td><td colspan="6">审查结果：
(1) 同意临床试验 ☑　　(2) 作必要的修改后同意 ☐
(3) 不同意临床试验 ☐　　(4) 终止或暂停已批准的试验 ☐
(5) 作必要修改后再审批 ☐
审查意见：

伦理委员会主任（签名）：[illegible]
2007 年 10 月 26 日</td></tr>
</table>

上海交通大学医学院附属仁济医院
伦理委员会出席成员名单

仁济伦审[2007] 56号

姓名	性别	职务	工作部门/职称	同意	不同意	签名
范关荣	男	主任	仁济医院院部、教授	同意		范关荣
李卫平	男	委员	仁济医院外科、教授	同意		李卫平
陆惠华	女	委员	仁济医院内科、教授	同意		陆惠华
钱家麒	男	委员	仁济医院内科、教授	同意		钱家麒
张 滨	男	委员	康昕律师所，律师	同意		张滨
孔宪明	男	委员	仁济医院科研部、教授	同意		孔宪明
王育	女	委员兼秘书	仁济医院政工部、副教授	同意		王育

表决结果

投票人数	7	同 意	7	反 对	0	弃权或回避	0

经医院伦理委员会的审阅和讨论，伦理委员会认为该项目符合医学伦理学原则和《药物临床试验管理规范》，请按照批准的研究方案进行临床试验，并定期提供研究进展报告。

记录： 王育　　　审批时间： 2007 年10月26日

地址：上海市东方路1630号(200127)．电话：(021)58752345－3417．传真：(021)58393018
