# Supplementary material for: Validation of Ten Noninvasive Diagnostic Models for Prediction of Liver Fibrosis in Patients with Chronic Hepatitis B
Source: PLoS One. 2015 Dec 28;10(12):e0144425. doi: 10.1371/journal.pone.0144425 (PMC4692502; doi:10.1371/journal.pone.0144425)
Supplement: S1 Appendix — (ZIP) [file pone.0144425.s001.zip › Ethics consent-302 Hospital of the China.pdf]

解放军第三〇二医院医学伦理委员会药物临床试验审批件

编号：医伦[2007]第 013 号

药物临床试验项目名称	FIBROSCAN 无创评价慢性乙型肝炎肝纤维化及肝硬化程度临床研究		
试验药物名称	中文名	—	
	英文名	—	
药物类别	—	SFDA 批件号	—
申办者	解放军第三〇二医院		
CRO	—		
临床试验专业	感染七科、病理科	项目负责人	陈菊梅
审阅文件	临床研究批件☑ 临床试验方案☑ 研究者手册☑		
	知情同意书 ☑ 药检报告 ☑ 研究者组成及简历☑		
医学伦理委员会审批意见			
同意 ☑		作必要的修正后同意 ☐	
不同意 ☐		终止或暂停已批准的试验 ☐	
医学伦理委员会审批说明			

本临床研究方案设计合理，同意开展此项临床研究，同时在试验中应充分做到知情同意，并注意受试者不良事件（尤其是肝穿相关不良事件）的发生，及时处理，保护受试者身体健康。

主任委员：陈菊梅

07年 9 月 13 日

解放军三〇二医院 医学伦理委员会
