# Supplementary figures and images for: Validation of Ten Noninvasive Diagnostic Models for Prediction of Liver Fibrosis in Patients with Chronic Hepatitis B
Source: PLoS One. 2015 Dec 28;10(12):e0144425. doi: 10.1371/journal.pone.0144425 (PMC4692502; doi:10.1371/journal.pone.0144425)

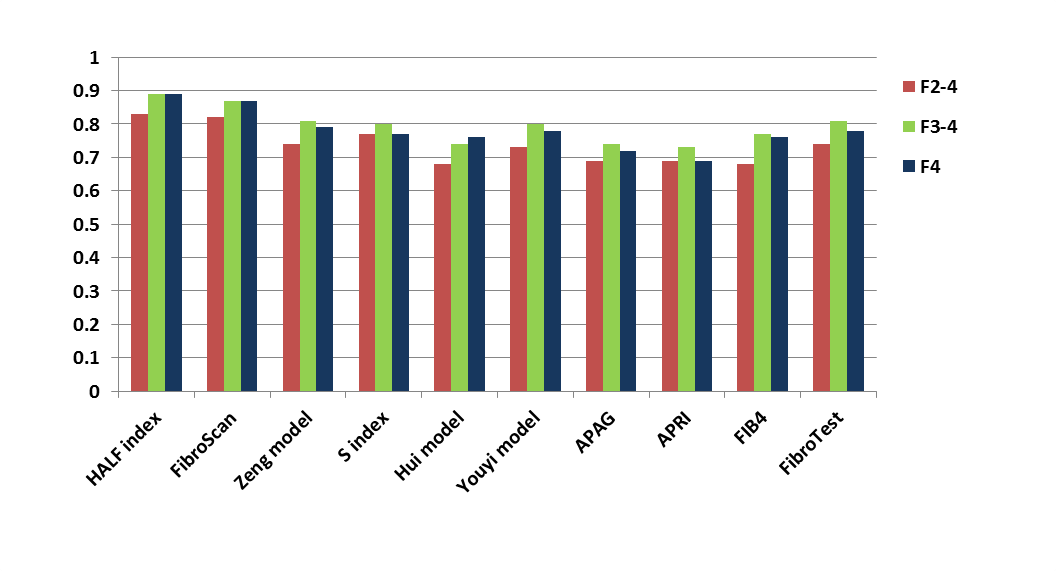

Supplement: S1 Fig — (TIF) [file pone.0144425.s003.tif]
