# Supplementary material for: Validation of Ten Noninvasive Diagnostic Models for Prediction of Liver Fibrosis in Patients with Chronic Hepatitis B
Source: PLoS One. 2015 Dec 28;10(12):e0144425. doi: 10.1371/journal.pone.0144425 (PMC4692502; doi:10.1371/journal.pone.0144425)
Supplement: S1 Table — (DOCX) [file pone.0144425.s004.docx]

**S1 Table.** **AUROC of respective models was compared with each other and the *P* values was listed as follow.**

| *AUROC For F2* | *FibroScan* | *Zeng model* | *S index* | *Hui model* | *Youyi model* | *APAG* | | *APRI* | | *FIB-4* | *FibroTest* | |
| --- | --- | --- | --- | --- | --- | --- | --- | --- | --- | --- | --- | --- |
| HALF index | 0.25 | <0.05 | <0.05 | <0.05 | <0.05 | <0.05 | <0.05 | | <0.05 | | | <0.05 |
| FibroScan | - | <0.05 | <0.05 | <0.05 | <0.05 | <0.05 | <0.05 | | <0.05 | | | <0.05 |
| Zeng model | - | - | 0.18 | <0.05 | 0.12 | 0.10 | 0.28 | | <0.05 | | | 0.87 |
| S index | - | - | - | <0.05 | <0.05 | <0.05 | 0.05 | | <0.05 | | | 0.27 |
| Hui model | - | - | - | - | <0.05 | 0.49 | 0.22 | | 0.80 | | | <0.05 |
| Youyi model | - | - | - | - | - | 0.40 | 0.66 | | <0.05 | | | 0.54 |
| APAG | - | - | - | - | - | - | 0.67 | | 0.42 | | | 0.18 |
| APRI | - | - | - | - | - | - | - | | 0.12 | | | 0.37 |
| FIB-4 | - | - | - | - | - | - | - | | - | | | <0.05 |
| *AUROC For F3* | ***FibroScan*** | ***Zeng model*** | ***S index*** | ***Hui model*** | ***Youyi model*** | ***APAG*** | | ***APRI*** | | ***FIB-4*** | ***FibroTest*** | |
| HALF index | 0.30 | <0.05 | <0.05 | <0.05 | <0.05 | <0.05 | <0.05 | | <0.05 | | | <0.05 |
| FibroScan | - | <0.05 | <0.05 | <0.05 | <0.05 | <0.05 | <0.05 | | <0.05 | | | <0.05 |
| Zeng model | - | - | 0.40 | <0.05 | 0.25 | <0.05 | <0.05 | | 0.15 | | | 0.95 |
| S index | - | - | - | <0.05 | 0.84 | <0.05 | <0.05 | | 0.28 | | | 0.74 |
| Hui model | - | - | - | - | <0.05 | 0.97 | 0.34 | | 0.48 | | | <0.05 |
| Youyi model | - | - | - | - | - | <0.05 | <0.05 | | 0.20 | | | 0.67 |
| APAG | - | - | - | - | - | - | 0.55 | | 0.34 | | | <0.05 |
| APRI | - | - | - | - | - | - | - | | 0.06 | | | <0.05 |
| FIB-4 | - | - | - | - | - | - | - | | - | | | 0.14 |
| *AUROC For F4* | ***FibroScan*** | ***Zeng model*** | ***S index*** | ***Hui model*** | ***Youyi model*** | ***APAG*** | | ***APRI*** | | ***FIB-4*** | ***FibroTest*** | |
| HALF index | 0.45 | <0.05 | <0.05 | <0.05 | <0.05 | <0.05 | <0.05 | | <0.05 | | | <0.05 |
| FibroScan | - | <0.05 | <0.05 | <0.05 | <0.05 | <0.05 | <0.05 | | <0.05 | | | <0.05 |
| Zeng model | - | - | 0.22 | 0.32 | 0.80 | <0.05 | <0.05 | | 0.21 | | | 0.53 |
| S index | - | - | - | 0.96 | 0.17 | <0.05 | <0.05 | | 0.65 | | | 0.87 |
| Hui model | - | - | - | - | 0.27 | 0.17 | <0.05 | | 0.69 | | | 0.77 |
| Youyi model | - | - | - | - | - | <0.05 | <0.05 | | 0.18 | | | 0.56 |
| APAG | - | - | - | - | - | - | 0.13 | | 0.39 | | | 0.21 |
| APRI | - | - | - | - | - | - | - | | <0.05 | | | <0.05 |
| FIB-4 | - | - | - | - | - | - | - | | - | | | 0.51 |
